# Probing Macromolecular Interactions by Loss of Solvent Content in Protein Crystals: Application to the Amyloid‑β Peptide Binding to Transthyretin

**DOI:** 10.1021/acs.cgd.5c00336

**Published:** 2025-05-07

**Authors:** Diogo Costa-Rodrigues, José P. Leite, Luís Gales

**Affiliations:** † ICBAS − Instituto de Ciências Biomédicas Abel Salazar, Universidade de Porto, 4050-313, Porto Portugal; ‡ i3S − Instituto de Investigação e Inovação em Saúde, 4200-135 Porto, Portugal; § IBMC - Instituto de Biologia Molecular e Celular, 4200-135 Porto, Portugal

## Abstract

X-ray crystallography is commonly used to determine the structure of protein–protein complexes, revealing the atomic details of the interactions between macromolecules in the crystal. However, this technique has limited application in binary systems characterized by transient or weak interactions. Here, we demonstrate that protein crystals can still provide valuable information in such systems by assessing crystal solvent content. We applied this method to investigate the interaction between transthyretin (TTR) and the amyloid beta (Aβ) peptide, a system of interest due to transthyretin’s proposed role in the clearance of Aβ peptide, whose accumulation in the brain is associated with Alzheimer’s disease. Soaking TTR crystals separately with Aβ fragments results in distinct reductions of the crystal void volume and highlights the key sequence residues involved in binding to TTR. Our findings indicate that the middle Aβ_12–28_ fragment interacts more strongly with TTR than the N- and C-terminals. Analysis of the crystal packing and solvent content indicates an equimolar interaction between transthyretin and the Aβ_12–28_ peptide. This interaction likely involves surface-exposed regions of TTR, such as the thyroxine binding pocket or the dimer pocket.

## Graphical abstract

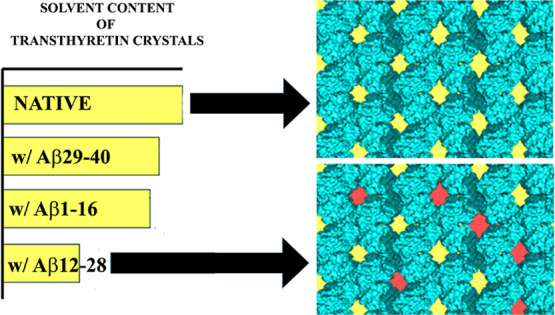

## 1 Introduction

Amyloid beta peptides (Aβ) are the main component of the amyloid aggregates found in the brains of patients with Alzheimer’s disease. The most abundant amyloid beta peptide is Aβ40 (referring to the 40 peptide residues), while the most reactive isoform is Aβ42. The concentration of Aβ42 in the cerebrospinal fluid (CSF) drops years or decades before clinical manifestations, presumably due to self-aggregation.^1–3^ Several research lines are being explored for treatment, primarily focused on antiamyloid approaches. These approaches include the reduction of Aβ production by inhibiting the two proteases, γ-secretase and β-secretase, involved in the release of this peptide fragment after the double cleavage of the amyloid precursor protein, disruption of the amyloid cascade by delivering active compounds, and promoting Aβ clearance. Aβ clearance using antiamyloid-β monoclonal antibodies has been extensively attempted with disappointing results.^4^ However, one antibody, aducanumab, was approved in a controversial decision by the Food and Drug Administration (FDA).

Aβ peptides can be eliminated through proteolytic or nonproteolytic pathways. Proteolysis is carried out by the so-called amyloid-degrading enzymes (ADEs), such as zinc–metalloproteases,^5,6^ serine proteases^7^ and cysteine proteases.^8^ Transthyretin (TTR) has been proposed as one of the major players in the regulation of Aβ levels in the CSF.^9,10^ It is not clear whether TTR sequesters Aβ into stable complexes^10^ or catalyzes the proteolytic degradation of Aβ.^11^ However, the binding of small compounds in the TTR channel stabilizes the protein,^12^ leading to a more compact quaternary structure^13^ and enhanced Aβ clearance activity.^14,15^

Despite the biochemical evidence, structural insights into the interaction between transthyretin and the amyloid-beta peptide remain elusive. The exception is a saturation transfer difference (STD) NMR analysis that was ingeniously used to investigate the nature of this interaction.^16^ TTR and Aβ seem to establish transient and/or weak interactions that hamper, at least in our hands, the determination of the crystal structure of the TTR/Aβ complex. Moreover, there are no structural models of this complex publicly available in the Protein Data Bank. When strong interactions are established with Aβ fragments, such as those involving certain antibodies and enzymes like thermolysin, the crystal structures of the resulting complexes can be determined.^17–25^

Here, we show that protein crystals, besides their conventional application in X-ray diffraction, may be used to investigate labile protein–ligand interactions. A few researchers have been calling attention to protein crystals as microporous materials that offer a wide range of pore size, porosity (0.3–0.9), and pore surface area (800–2000 m^2^ g^–1^).^26,27^ Recently, we used cross-linked protein crystals as molecular sieves for the extraction of monomeric TTR from the plasma, which, combined with a subsequent immunodetection step, enabled us to devise an assay for sensing circulating monomeric TTR and establish this target as a biomarker of TTR amyloidosis.^28^ The assay was then adapted to evaluate TTR stabilizer candidates using plasma samples.^29^ In this work, we incubated TTR crystals separately with Aβ fragments spanning the full peptide sequence and observed significant differences in the remaining free crystal void volumes, reflecting how each fragment populated the crystal solvent volume and interacted with the host TTR protein. To assess the decrease in void volume, crystals were soaked in a fluorescent probe both before and after incubation with Aβ fragments. The strategy is useful to identify the residue sequence of the Aβ peptide that plays a key role in the interaction with TTR.

## 2 Experimental Section

### 2.1 Production and Purification of Recombinant TTR

The recombinant TTR proteins were produced using a pET expression system (Novagen). Mutant proteins were generated by PCR site-directed mutagenesis using the QuickChange kit (Stratagene) and expressed in *Escherichia coli* BL21 (DE3) cells harboring the corresponding plasmid. Expression cultures in lysogeny broth (LB) medium containing 50 μg mL^–1^ kanamycin were grown at 37 °C to an optical density of 0.6 at 600 nm, then induced by the addition of isopropyl-β-D-thiogalactoside (1 mM final concentration), grown at 37 °C for 20 h, and harvested by centrifugation (13,700*g* for 15 min).

After cell lysis by sonication, intracellular proteins were fractionated by ammonium sulfate precipitation. The TTR-containing fraction precipitated between 55% and 85% ammonium sulfate saturation. The precipitate was dissolved in 20 mM Tris (pH 7.2) with 0.1 M NaCl and dialyzed against the same buffer. It was then applied to a Q-Sepharose High-Performance anion exchange column (Amersham Biosciences) and eluted using a linear gradient of 0.1–0.5 M NaCl in 20 mM Tris (pH 7.2). TTR-enriched fractions were dialyzed against 5 mM Tris (pH 7.2) with 2.5 mM NaCl, lyophilized, and redissolved in a small volume of buffer (10 mM Tris, pH 7.2, 0.1 mM NaCl).

Further purification was performed using gel filtration chromatography on a Superdex 75 prep-grade column (Amersham Biosciences), with elution in 10 mM Tris (pH 7.2) containing 0.1 M NaCl. The purest fractions were combined, dialyzed against 20 mM phosphate buffer (pH 7.6) with 100 mM KCl, and stored at 4 °C. Protein concentration was determined spectrophotometrically at 280 nm using an extinction coefficient of 77,600 M^–1^ cm^–1^.

### 2.2 TTR Crystallization and Cross-Linking (TTR-CLC)

Crystals were obtained by hanging-drop vapor-diffusion techniques at 20 °C according to the method described in.^30–32^ Crystals were grown within 1 week by mixing 2 μL of the protein solutions with 2 μL of reservoir solutions. The reservoir solutions used in the crystallization trials contained acetate buffer 0.2 M pH 4.8–5.4, ammonium sulfate 1.8–2.2 M, 7% glycerol. TTR crystals were cross-linked (TTR-CLC) by adding one drop of 1 μL of 25% v/v glutaraldehyde directly to the crystal’s drop. The well was then closed again (well-sealed with grease to avoid vaporization of glutaraldehyde), and the crystals were left incubating for 1.5 h.

### 2.3 Aβ Peptides

Aβ_1–16_, Aβ_12–28_, Aβ_29–40_ (subscript numbers indicate residue number) were purchased from Bachem (Switzerland), with a sample purity of 93.7, 99.2 and 98.9%, respectively, as determined by HPLC in trifluoroacetic acid by the supplier. Amino acid sequences are as follows: DAEFRHDSGYEVHHQK for Aβ_1–16_ (*M*_W_ 1955.0), VHHQKLVFFAEDVGSNK for Aβ_12–28_ (*M*_W_ 1955.2), and GAIIGLMVGGVV for Aβ_29–40_ (*M*_W_ 1085.4). Aβ samples were prepared based on the ref 33. Briefly, lyophilized Aβ peptides were equilibrated at room temperature for 30 min to allow complete defrosting and spun before opening the vial, to maximize the sample retrieval, and then dissolved in 1,1,1,3,3,3-hexafluoro-2-propanol (HFIP) and incubated at room temperature for 3 h to disassemble possible pre-existing amyloid aggregates. At this stage, samples should be clear. If a suspension was formed, samples were briefly sonicated in a water bath, HFIP was removed by overnight evaporation in a fume hood and further dried with a vacuum concentrator for 2.5 h to remove remaining traces. Dried peptide films were thoroughly resuspended in dimethyl sulfoxide (DMSO), and concentrations of the stock solutions were determined using the Pierce BCA Protein Assay Kit (Thermo Fisher Scientific, USA) using bovine serum albumin as standard and multiplied by a 1.51 conversion factor that accounts for the different chromophoric development of albumin and Aβ peptides.

### 2.4 TTR-CLC Incubation with Aβ Fragments and Estimation of the Crystal’s Solvent Content

TTR-CLC crystals were incubated separately with three Aβ fragments that span the entire peptide sequence: Aβ_1–16_ (*M*_W_ 1955.0), Aβ_12–28_ (*M*_W_ 1955.2), and Aβ_29–40_ (*M*_W_ 1085.4). A measurable reduction in the crystals’ free porous volumes is anticipated if one of the guest peptides exhibits a high affinity for the TTR molecules within the crystal lattice. This void is subsequently estimated by the uptake of fluorescein, which is suitable for optical sensing due to its fluorescence intensity, allowing for precise quantification of the uptake extent. This experimental procedure, schematically illustrated in Figure 1, is adapted from the approach used to estimate the uptake of monomeric TTR by cross-linked protein crystals.^29^

**Figure 1:**
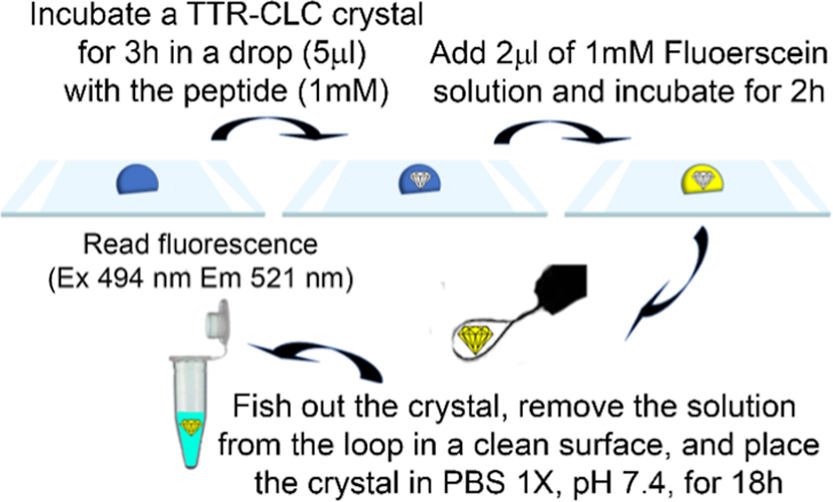
Scheme of the experimental procedure used for TTR-CLC incubation with Aβ fragments and estimation of the crystal’s solvent content.

Briefly, TTR-CLC single crystals are incubated for 3 h in a 5 μL drop with an Aβ peptide. Then, 2 μL of a solution of 1 mM fluorescein is added to the crystal drop and incubated for 2 h. The crystals are then fished out using a nylon loop, gently placed in a clean surface to remove the solution in the loop and transferred, also with a loop, to a 100 μL phosphate buffered saline solution. The fluorescence of these fluorescein-releasing solutions is measured after 18 h (excitation at 494 nm and emission at 521 nm). The incubation times were initially screened to allow the system to reach equilibrium at each step. Preincubation with amyloid beta fragments delayed fluorescein equilibration from 30 min to 2 h. The final step was carried out overnight for practical reasons, taking additional time.

### 2.5 Crystal Structure Determination of TTR Incubated with Aβ Fragments

The three fragments—Aβ_1–16_, Aβ_12–28_, and Aβ_29–40_—prepared in stock DMSO solutions were added in one attempt to TTR solutions (10 mg.mL^–1^ in 10 mM HEPES, pH 7.5) to achieve a final DMSO concentration of 5%. The solutions were incubated overnight, and cocrystallization was performed as described in Section 2.2.

In another attempt, TTR crystals were first grown as described in Section 2.2. The Aβ peptide stock solutions were then added to the crystal drops (final DMSO concentration <10%) and incubated for 30 min.

Crystals were transferred to reservoir solutions containing increasing concentrations of glycerol (10–20%) and flash-frozen in liquid nitrogen. X-ray diffraction data were collected using synchrotron radiation at the ESRF (European Synchrotron Radiation Facility, Grenoble Cedex, France), ALBA (Barcelona, Spain), and SOLEIL (Paris, France).

The software package Phenix^34^ was used and molecular replacement carried with PhaserMR.^35^ TTR crystal structure (PDB accession no. 1Y1D^31^), after the removal of water molecules and the ligand, was used as the starting model. The final models were obtained after multiple cycles of refinement and manual model building, carried out with Phenix.refine^36^ and Coot,^37^ respectively.

### 2.6 TTR Hydrolysis of the Aβ_12–17_ Fragment

The fluorogenic Aβ_12–17_ derivative Abz-VHHQKL-EDDnp was purchased from Genscript (USA) and reconstituted at 1 mM in 50% DMSO. Hydrolysis in the Aβ region results in the separation of the fluorescent donor Abz (*ortho*-aminobenzoic acid) from the fluorescent quencher EDDnp [*N*-(ethylenediamine)-2,4-dinitrophenyl amide], leading to an increase in fluorescence. TTR-catalyzed hydrolysis was monitored by continuous fluorescence measurement (excitation at 320 nm; emission at 420 nm) at 37 °C, every 4 min, using a Synergy Mx (BioTek, USA). To prevent evaporation, plate wells were sealed with paraffin oil. Triplicate measurements were performed in 50 mM Tris–HCl, pH 7.4, with five different TTR concentrations: 0.05, 0.1, 0.25, 0.5, and 1 μM.

### 2.7 Statistical Analysis

Three independent experiments were performed for each Aβ fragment. Data were analyzed using GraphPad Prism Software (version 9.5.0). An unpaired *t*-test with Welch’s correction was performed. Levels of statistical significance at * *p* < 0.05, ** *p* < 0.01, *** *p* < 0.001, **** *p* < 0.0001 were used.

## 3 Results and Discussion

### 3.1 Uptake of Aβ_1–16_, Aβ_12–28_, and Aβ_29–40_ by TTR Crystals

TTR crystals were grown using the vapor diffusion method. After each crystallization batch, a single crystal was selected for X-ray diffraction data collection using our in-house X-ray diffractometer to determine the unit cell dimensions and lattice type. The crystals were consistently orthorhombic, belonging to space group *P*2_1_2_1_2, and were cross-linked to enhance stability, allowing for easier manipulation and preventing dissolution during multiple transfer steps. The experimental data on the porosity of cross-linked protein crystals are usually in good agreement with theorical estimation content based on crystallographic information.^26^

We have already determined the structure of TTR crystals, which are isomorphous to those used in the soaking experiments, using synchrotron radiation.^14,31,32,38,39^ The packing reveals a solvent content of 41%, with *a*-channels running along the *a* crystallographic axis, featuring a bottleneck radius of 8.5 Å^40^ (Figure 2).

**Figure 2:**
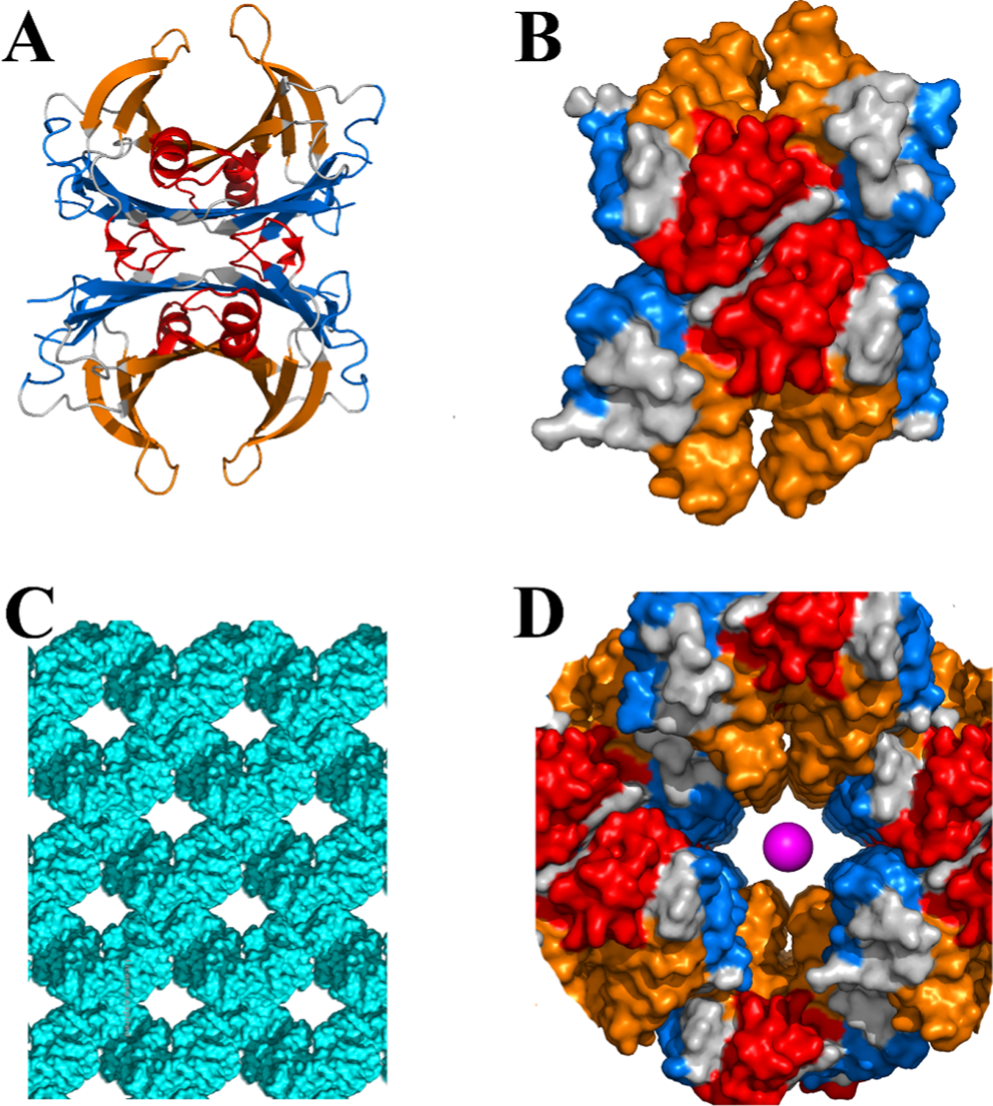
Structural features of TTR and crystal packing. Cartoon (A) and surface (B) representations of TTR highlighting the T4 binding pocket (blue), the RBP binding pocket (red) and the dimer pocket (orange). (C) Crystal packing along the *a*-axis, illustrating the crystal channels. (D) Close-up view of a crystal channel, emphasizing the exposed TTR surface regions. The magenta sphere has a radius of 4.5 Å, which corresponds to the approximate hydrodynamic radius of fluorescein. Note: (B–D) are not represented at the same scale.

The effectiveness and sensitivity of the proposed method for probing intermolecular interactions is intrinsically linked to the crystallographic packing characteristics of the host protein, the exposure of its interaction surfaces, and the size of the guest protein or peptide. Guest biomolecules must be sufficiently large to significantly reduce the crystal’s solvent content yet small enough to diffuse through the crystal framework. A fluorescent probe is used to assess the crystal solvent content both before and after incubation with the guest. Typically, a guest macromolecule cannot access the full internal volume of the crystal. To ensure maximal sensitivity to changes in guest molecule occupancy, it is ideal to use a probe that samples the same crystal void volume as the guest itself.

In this study, fluorescein was used as a molecular probe. Automated soakability predictions of the TTR crystals, performed using the LifeSoaks program,^40^ indicate that fluorescein—with a hydrodynamic radius of approximately 4.5 Å—is excluded from the narrower channels within the crystal and can only penetrate the same regions accessible to the sampled Aβ fragments—the *a*-channels. The *a*-channels have a periodicity of ∼43 Å, matching the unit cell length along the *a*-axis, with each periodic segment containing one TTR tetramer. Because of their restricted dimensions (bottleneck radius of 8.5 Å), the narrow channels exclude fluorescein from occupying the same segments as the Aβ fragments. For instance, the Aβ_12–28_ peptide, which has a hydrodynamic radius of approximately 11 Å,^41^ is expected to align along the *a*-channel axis. Assuming a fully aligned orientation of the Aβ fragment with the crystallographic *a*-axis allows for a slight overestimation estimation of the reduction in fluorescein uptake for a given TTR/Aβ fragment stoichiometry within the crystals. For example, two Aβ_12–28_ molecules per TTR tetramer would fully occupy the channel, thereby excluding fluorescein entirely, while a single Aβ_12–28_ molecule would reduce fluorescein uptake by approximately half.

The analysis of the regions of transthyretin (TTR) exposed on the surface of crystal channels can provide insights into its molecular interaction processes. TTR possesses several binding sites, notably the thyroxine (T4) binding pocket, the retinol-binding protein (RBP) binding pocket, and an additional dimer pocket, as illustrated in Figure 2. Due to the conformational constraints within the crystal lattice, only the T4 binding pocket and the dimer pocket are exposed to the crystal channels, making them accessible for potential interactions Aβ_12–28_ fragments. Full saturation of T4 binding sites can be achieved by soaking TTR crystals in ligand solutions, demonstrating that the T4 binding pockets remain accessible in the crystals.^42^

TTR-CLC crystals were incubated separately with three Aβ fragments that span the entire peptide sequence: Aβ_1–16_ (*M*_W_ 1955.0), Aβ_12–28_ (*M*_W_ 1955.2), and Aβ_29–40_ (*M*_W_ 1085.4).

By measuring fluorescein uptake in the same TTR-CLC crystal before and after incubation with the Aβ fragment, we can estimate the fraction of the original fluorescein-accessible void volume that becomes occupied by the Aβ fragment. This approach mitigates uncertainties related to determining the crystal volume and to the slight reductions in crystal porosity induced by cross-linking.

The uptake and release of fluorescein from TTR-CLC not preincubated with Aβ fragments occur relatively quickly (within 30 min), suggesting a lack of strong binding between the probe and the protein framework. Given that the isoelectric point of transthyretin (TTR) is 5.5 and fluorescein carries a net charge of −2 at pH 7.4, electrostatic interactions are unlikely to contribute significantly to binding. Interactions between fluorescein and proteins have been more extensively studied using lysozyme as a model. Despite lysozyme having a net positive charge at neutral pH, its maximum adsorption capacity for fluorescein within the crystal structure remains modest—approximately 1 mol of fluorescein per mol of lysozyme.^43^

The results for the three Aβ fragments are presented in Figure 3. The three Aβ fragments occupy the TTR-CLC framework up to measurable extents. However, the Aβ_12–28_ fragment shows a significantly higher occupation rate. It is important to note that the molecular weight (*M*_W_) of Aβ_29–40_ is significantly lower than that of the other two fragments, that display similar *M*_W_s. Even after adjusting for these *M*_W_ differences, as indicated by the lighter shade of pink in Figure 3, this fragment still exhibits the lowest molar incorporation extent.

**Figure 3:**
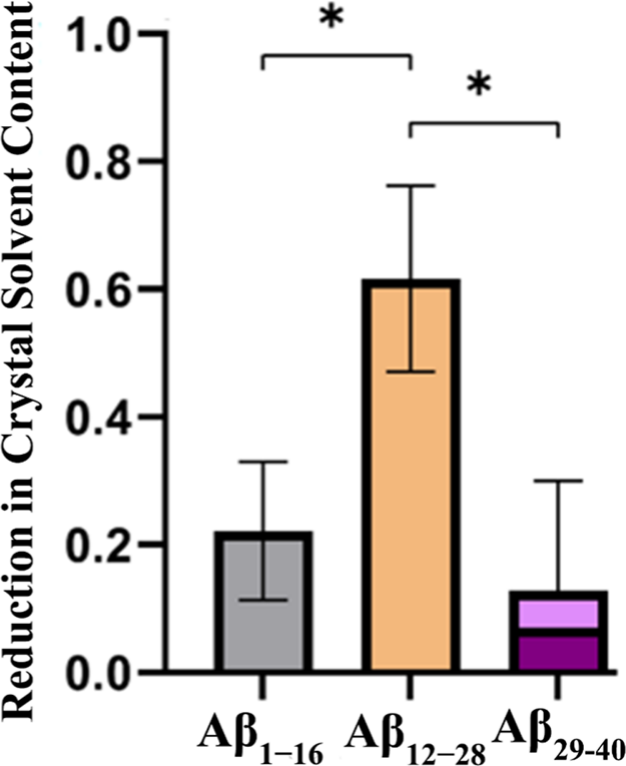
Fractional reduction of TTR crystal solvent content after soaking with Aβ fragments. TTR crystals internalize Aβ_12–28_ in higher amounts than the other fragments. For Aβ_29–40_, a correction accounting for its smaller molecular volume compared to the other two Aβ fragments is shown in pink. Aβ_1–16_ and Aβ_12–28_ have similar molecular sizes.

Accordingly to the initial analysis of the expected correlation between the TTR: Aβ_12–28_ stoichiometry and the fractional reduction of the TTR crystal solvent content estimated using the fluorescein probe the results are consistent with the presence of one Aβ_12–28_ molecule per TTR tetramer. The 2-fold symmetry of the crystal channels, limited by two opposite T4-binding pockets and two opposite dimer pockets, suggests that there are two symmetry-related overlapping binding positions for Aβ_12–28_. Thus, in solution, free from the crystallographic constraints, it is likely that two Aβ_12–28_ molecules may interact simultaneously with a TTR tetramer. On the other hand, diffusional restrictions and the repetitive arrangement of TTR subunits may contribute to an increased frequency of TTR–Aβ contacts in the crystal environment compared to solution.

### 3.2 The Importance of the Diphenylalanine Motif in the Binding of Aβ_12–28_ to TTR Crystals

The Aβ_12–28_ fragment contains the diphenylalanine (FF) motif at positions 19 and 20, a sequence frequently associated with the propensity of Aβ to self-assemble into amyloid aggregates. Given its relevance in aggregation, we sought to investigate whether this motif also plays a role in the interaction with TTR.

Probing this interaction using our method is challenging due to the small volume of the FF dipeptide. As depicted in Figure 4, the presence of the FF dipeptide alone does not appear to significantly affect the total void volume of the crystal. This suggests that although the dipeptide may be capable of binding within the crystal channels, it does not substantially alter the overall solvent content.

**Figure 4:**
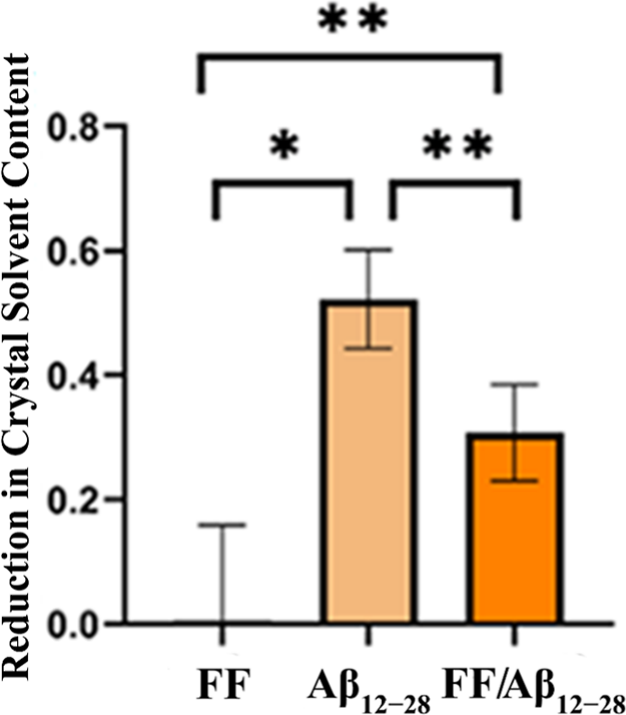
Fractional reduction of TTR crystal solvent content after soaking. Crystals soaked with FF, with Aβ_12–28_ (salmon), and sequentially soaked with FF and Aβ_12–28_ (orange).

However, when TTR crystals are preincubated with the FF dipeptide and subsequently soaked with the Aβ_12–28_ fragment, they exhibit an intermediate solvent content. This solvent content is higher than that observed in crystals directly incubated with Aβ_12–28_ but lower than that of native crystals or those incubated with N- or C-terminal Aβ fragments. This observation suggests competitive binding between the FF dipeptide and the Aβ_12–28_ fragment, leading to a partial reduction in Aβ_12–28_ occupancy within the TTR crystal channels.

### 3.3 Crystal Structures of TTR with Aβ Fragments

Crystals obtained from both cocrystallization and soaking of TTR with the three Aβ fragments consistently exhibited the space group *P*2_1_2_1_2, with cell dimensions comparable to those of native TTR crystals. Despite thorough data processing and refinement, no evidence of Aβ fragment incorporation was detected (data not shown), including Aβ_12–28_, which had previously shown promising interaction results. This absence highlights the transient and potentially dynamic nature of their interactions, suggesting that Aβ binding to TTR may be weak, unstable, or influenced by conformational flexibility, preventing stable incorporation into the crystal lattice.

### 3.4 TTR-Mediated Hydrolysis of the Aβ_12–17_ Fragment

The Aβ_12–28_ fragment exhibits a stronger interaction with TTR compared to the N- and C-terminal fragments, likely due to the presence of the diphenylalanine motif, which appears to enhance the binding affinity of the central region. To further explore this, we will now examine TTR’s interaction with the Aβ_12–17_ fragment, which lacks the diphenylalanine sequence, with particular attention to TTR’s reported hydrolytic activity toward this fragment.^44^

We analyzed the hydrolysis of the fluorogenic Aβ_12–17_ derivative Abz-VHHQKL-EDDnp. TTR was found to hydrolyze this derivative in a concentration-dependent manner, with reaction curves (Figure 5) fitting well to the Michaelis–Menten equation, yielding a *K*_cat_/*K*_m_ value of 4.7 × 10^2^ M^–1^ s^–1^. Using the same assay, we determined a *K*_cat_/*K*_m_ value of 9.3 × 10^3^ M^–1^ s^–1^ for neprilysin-mediated degradation of the same substrate.^33^ This suggests that TTR either exhibits low substrate-binding affinity toward the Aβ_12–28_ fragment or slow catalytic activity.

**Figure 5:**
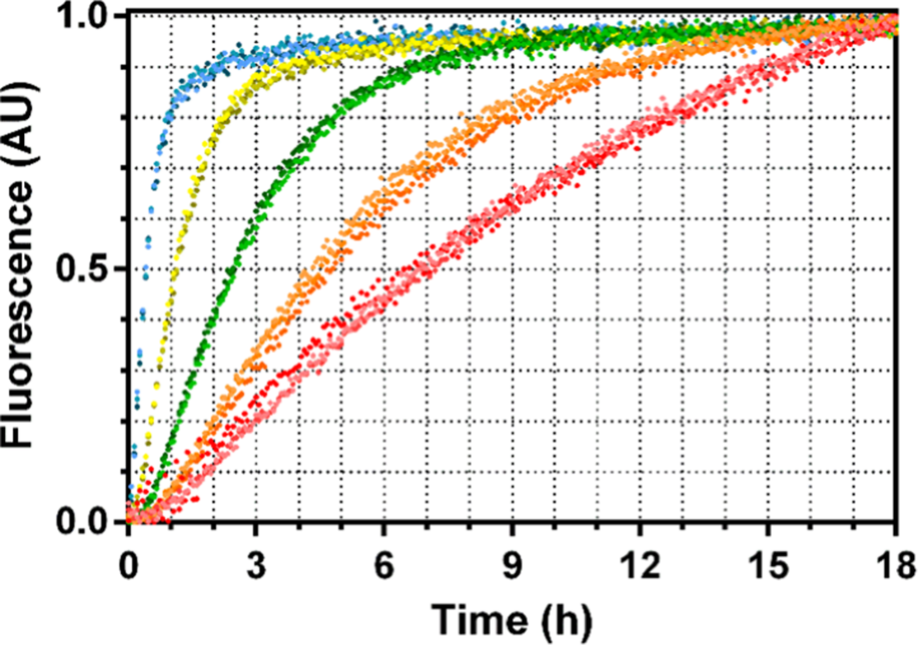
TTR hydrolyses Abz-VHHQKL-EDDnp. Hydrolysis of 5 μM Abz-VHHQKL-EDDnp by TTR in a range of proteins concentrations: 0.05 μM (red), 0.1 μM (orange), 0.25 μM (green), 0.5 μM (yellow) and 1 μM (blue). Triplicate measurements for each protein concentration are indicated by dots in shades of the same color. Fluorescence (A.U.) normalized to fractional scale.

Even considering the low reaction rate and the much higher ratio of Aβ relative to TTR used in the previous crystal soaking experiments, it is possible that, in the case of TTR crystals incubated with the Aβ_12–28_ fragment, the crystal void volume was occupied not only by Aβ_12–28_ but also, to a lesser extent, by its proteolytic products.

## 4 Conclusions

Our study highlights the potential of protein crystals to probe transient macromolecular interactions by assessing changes in solvent accessibility. Using cross-linked TTR crystals, we demonstrated that different Aβ fragments interact with TTR to varying degrees, with the Aβ_12–28_ fragment showing the highest affinity. Moreover, our experiments suggest that the presence of the FF dipeptide influences the extent of Aβ_12–28_ binding.

Analysis of the crystal packing and solvent content reveals an equimolar interaction between transthyretin (TTR) and the Aβ_12–28_ peptide. This interaction likely involves surface-exposed regions of TTR, such as the T4-binding pocket or the dimer interface. Despite these promising results, cocrystallization and soaking experiments did not reveal direct electron density for Aβ fragments in TTR crystal structures, highlighting the transient nature of their interactions. Nevertheless, our findings align with insights into TTR-Aβ interactions in solution obtained through Saturation Transfer Difference NMR analysis.^16^ The use of fluorescein uptake proved to be an effective method for mapping interaction sites in weakly associating systems. Our approach provides a valuable tool for studying elusive protein–protein interactions and could be applied to other biological systems where direct structural determination remains challenging.

These findings contribute to the understanding of TTR’s role in Aβ clearance and may inform future therapeutic strategies targeting Aβ accumulation in Alzheimer’s disease. Further studies are needed to explore the mechanistic implications of TTR-mediated Aβ binding.

## Abbreviations

Abz: *ortho*-aminobenzoic acid
Aβ: amyloid beta peptide
DMSO: dimethyl sulfoxide
EDDnp: [*N*-(ethylenediamine)-2,4-dinitrophenyl amide]
RBP: retinol binding protein
PDB: protein data bank
T4: thyroxine
TTR: transthyretin
TTR-CLC: transthyretin cross-linked crystals.
